# Predicting risk of avian influenza a(H5N1) in Egypt: the creation of a community level metric

**DOI:** 10.1186/s12889-018-5288-x

**Published:** 2018-03-21

**Authors:** Ellen C. L. Geerlings, Claire Heffernan

**Affiliations:** 1 Department of Agriculture, University of Reading, Reading, UK; 2 School of Veterinary Sciences, University of Bristol, Bristol, UK; 3 Research & International Development Consultancy Services (EGRID), Deventer, The Netherlands; 4 London International Development Centre (LIDC), London, UK

**Keywords:** Egypt, Influenza a virus, H5N1 subtype, Public health, Risk factors, Decision support techniques

## Abstract

**Background:**

Efficient A(H5N1) control is unlikely to be based on epidemiological data alone. Such control depends on a thorough understanding and appreciation of the interconnectedness of epidemiological, social, and economic factors that contribute to A(H5N1) vulnerability. To date, the control of A(H5N1) in Egypt has been challenging. The disease has been endemic for more than 10 years with a dramatic increase in human cases between December 2014 and March 2015. Part of the problem has been a lack of understanding of the inter-play of drivers, conditions and motives that influence preventive behaviours at the household level.

**Methods:**

To address this issue, the authors developed a Composite Risk Index (CRI) to inform decision-makers of critical epidemiological, livelihood, food security and risk perception factors that were found to contribute to A(H5N1) vulnerability at the community level. The CRI consists of seven constructs that were individually scored for each community. The seven constructs included poultry sales, previous flock exposure to A(H5N1), human risk probability, sense of control over the disease, preventative actions taken, level of household food insecurity and community norms toward certain handling and disposal practices. One hundred forty female poultry keepers across four governorates were interviewed in 2010 using a mix of random and purposive sampling techniques. A mixed method approach underpinned the analysis. The study used wealth ranking in order to help decision-makers in understanding the specific constraints of different wealth groups and aid better targeting of A(H5N1) control and prevention strategies.

**Results:**

Poverty, widowhood and lack of education were among the factors associated with high risk scores. CRI scores in those villages where awareness raising had taken place were not significantly different compared to those villages where awareness raising had not taken place.

**Conclusions:**

The aim of the tool is to enable targeting those communities that are likely to be highly vulnerable to A(H5N1) outbreaks and where control and awareness-raising efforts are expected to be most effective. In this manner, policy makers and practitioners will be able to better allocate limited resources to those communities most vulnerable to the negative impact of A(H5N1).

**Electronic supplementary material:**

The online version of this article (10.1186/s12889-018-5288-x) contains supplementary material, which is available to authorized users.

## Background

Highly Pathogenic Avian Influenza (HPAI) subtype H5N1, hereafter referred to as A(H5N1), has been endemic in Egypt for almost a decade. Up to 8 January 2018, 860 human cases have been reported worldwide, 42% of these cases have been reported in Egypt [1]. Over 90% of human infections are due to close contact with backyard poultry [2]. The majority of clinical cases have been women and children, who are the main caretakers of household flocks in rural areas [2–5].

It is estimated that five to seven million Egyptian rural households are involved in household poultry production [6]. Poultry meat is the most consumed animal protein source, it supplies roughly 53% of daily protein intake compared to bovine, mutton and goat meat [5, 7]. This share is likely higher among the poor as poultry meat is relatively cheap compared to other meats. Furthermore, poultry provide a relatively large share of total household income particularly among the poor and widowed [8, 9]. A flock of 73 birds (mixed species) provides an average annual net income of approximately US $397 [6]. This is significantly more than a government widower’s pension of approximately US $140 annually.

Historically, the government of Egypt has adopted a number of mechanisms aimed at limiting the spread of A(H5N1) and lowering the risk of human infection, with varying degrees of success. Traditional stamping out mechanisms such as widespread culling of flocks and mass vaccination have been heavily relied upon to control the disease [10–13]. Despite such measures, Egypt has experienced a dramatic increase in human cases during the winter of 2014/2015 [3, 12–15]. Nonetheless the number of human cases is likely underreported [2, 15]. Kayeli et al. [15] conclude from a serologic study that the true number would probably amount to several hundred thousand human cases.

Control efforts have also included awareness-raising campaigns at the community level to promote the application of basic biosecurity measures [5]. However, few baseline studies have been conducted, which would have given insight into risk behaviour of specific target groups including subsets of household poultry producers [10]. Although these campaigns succeeded in raising awareness, the problem of low uptake of these measures at the household level remained [5, 10, 16]. This phenomenon of high A(H5N1) awareness but low uptake of preventive measures has been reported in many other contexts [17–19]. Reasons for non-compliance with preventive measures are manifold. Mistrust in government practices play an important role, as well as a lack of information at the community level as reported for Egypt [5, 10, 11, 16] but also in Asia [18, 21, 22]. In Egypt post vaccination sudden death of poultry, likely caused by incorrect vaccination or lack of biosecurity practices on the part of veterinary personnel, caused reluctance to vaccinate among poultry owners [11]. In addition the vaccines used in the field were not efficacious due to improper antigenic matching between vaccines and circulating viruses [2, 15, 22].

Equally problematic, in recent years there has been a decline in public awareness of the risks [5, 10, 12, 14, 22]. Indeed, A(H5N1) outbreaks in poultry in Egypt are suspected to be heavily underreported [10, 23]. Women, in particular are often reluctant to report the disease mainly out of fear of loss of income, loss of poultry meat and eggs to feed the family and the inability to fulfil certain socio-cultural roles such as providing guests with poultry meat and eggs or contributing to particular cultural and religious ceremonies [8, 9]. Similarly, Paul et al. [20] describe how financial value and prestige combined with cultural value, fear of loss of genetic resources and personal bond of owners with their cocks all contributed towards reluctance to report outbreaks among the cockfighting community in Thailand. In Vietnam reporting was associated by farmers with uncertain outcomes and transaction costs, in addition to market disturbance resulting in lower market value of poultry [21].

Social norms may also prevent poultry keepers from applying biosecurity measures. In one study in Egypt a situation was described where participants explained that they had been mocked within their community for wearing protective clothes when dealing with poultry [16]. Social norm also plays an important role in influencing how cock farmers manage A(H5N1) infection risk among a tight-knight community in Thailand [20].

Therefore, a deeper understanding of the perceptions of risk, local beliefs and rationales regarding A(H5N1) and social norm are needed for understanding and facilitating changes in biosecurity behaviours [5, 20, 21, 24]. As Fielding et al. ([24]: page 19) clarified:

“*simply providing information takes no account of a population’s causal attributions, perceived risks, perceptual bias or structural determinants of behaviour, and is unlikely to result in significant and sustained change.”*

Egyptian government veterinary services are stretched in terms of human and financial resources [10, 11, 25]. Vaccination alone absorbed more than 80% of the budget for influenza control [10, 23]. Limited human resources and low vaccination coverage, coupled with improper use of vaccines and limited biosecurity precautions practiced by the vaccination squads have been a major contributory factor for the A(H5N1) endemic [10, 11, 23, 25].

Since the 2011 political crisis the situation has deteriorated. Unemployment remains persistently high, particularly among women [26]. Food prices have risen and poverty has increased particularly in rural Upper Egypt where 57% fall below the income poverty line [26]. At present the incidence of A(H5N1) and A(H9N2) is increasing and co-infection of poultry with these two viruses may create the potential for reassortment of these viruses [2, 15]. The present economic crisis is likely to increase people’s dependency on poultry as a source of income and a cheap source of animal protein. At the same time coping strategies aiming at preserving poultry as a source of income and food may involve risky practices. Media attention to A(H5N1) has dwindled and has moved on to more acute issues such as the economic and political crisis. This has led people to believe A(H5N1) no longer possess a threat and reduces the felt need to adhere to biosecurity practices that were already badly understood and perceived as cumbersome and impractical [5, 8, 16].

Much of the literature on A(H5N1) has taken an epidemiological approach describing and analysing (the often lack of) biosecurity practices of backyard poultry producers. However, there is an emerging body of literature describing the social context in which infectious and zoonotic disease risk is embedded [5, 20, 27–32]. A holistic approach including cultural, social, economic and epidemiological aspects must be part of a comprehensive risk assessment and control strategy [2, 14, 33]. Such an approach is more relevant now than ever as Egypt is regarded as a hotspot for the emergence of a pandemic potential virus while at the same time A(H5N1) awareness among those most at risk is at a low.

Given the complex mix of factors contributing to risk perceptions and behaviours among household poultry producers, and the limited resources of government veterinary services, there is a need for a more targeted approach to control and awareness-raising activities at the community level. To address this need, the authors created a Composite Risk Index (CRI). The CRI is based on a range of epidemiological, livelihood, food security and risk perception factors mentioned in the literature, which were found to contribute to vulnerability to A(H5N1) infection. In line with Blaikie et al. [34], vulnerability is understood as the potential to suffer harm or loss, related to the capacity of poultry producers to anticipate, cope with, and recover from, the impact of an A(H5N1) outbreak in their household flock. Vulnerability, and its opposite resilience, are determined by a mix of physical, social, economic, cultural, political and environmental factors [35, 36].

Asset portfolios influence risk attitudes as well as the means available to manage risk [37]. Earlier work [8, 9] indicated that the poor have been hardest hit by the impact of A(H5N1) outbreaks and subsequent culling and control efforts. Other authors have also described how the poor and uneducated were at higher risk of infection [18]. Wealthier households (in terms of asset base) are generally believed to be “*more efficient in resource allocation and better situated to handle risk-related losses*” ([37]: page 4). Therefore, the CRI distinguishes between subsets of the poor in order to help decision-makers in understanding the specific constraints of different wealth groups and aid better targeting of A(H5N1) control and prevention strategies.

## Methods

### The sample frame

The CRI was underpinned by data from 140 household interviews and 24 group discussions among female poultry keepers from lower socio-economic strata residing across four governorates of Egypt: Fayoum, Assuit, Menia and Sohag. The governorates were selected based on their high poverty levels, importance of poultry measured by the percentage of households keeping poultry and exposure to A(H5N1) outbreaks in backyard poultry flocks. The four selected governorates were ranked last by the Human Development Index at the time of the study [38]. A WFP/VAM study indicated that the proportion of households keeping poultry was highest in the governorates of Menia, Assuit, Souhag and Fayoum (WFP/VAM unit Egypt Country Office, unpublished data). Official reports indicated that all study districts and governorates had experienced outbreaks of A(H5N1) in 2006 and onwards [39].

Pilot testing of the questionnaires took place in August 2010 by the author with the help of a translator. Three days were spent in the field, testing key informant interviews, group discussions and household interviews. The questionnaires were tested in three different villages in Fayoum Governorate. Enumerators included two Egyptian female veterinarians with experience of working in rural areas and one Egyptian male agricultural engineer experienced in rural development. All enumerators underwent a three-day training course where a combination of classroom theory, practical fieldwork and evaluations were used to prepare for the fieldwork. The training had a particular focus on minimizing bias and encouraging open and frank discussions with study participants. Because of cultural and religious norms, the female enumerators were responsible for the individual interviews with female poultry keepers while the male enumerator would lead the introduction of the research team to the village head and other village leaders, as well as conducting the key informant interviews. Individual interviews took place at a location chosen by the interviewee. In the majority of cases, this would be the home or yard of the female poultry keepers and sometimes in front of their houses or in vegetable stalls or kiosks. Group interviews were often conducted with one male and one female enumerator. As the group interviews took place in public settings with more than one woman, the presence of a male enumerator was not an issue. The questionnaires used for this study can be found in Additional file 1.

Two districts within each of the above-mentioned governorates, and three villages in each district were randomly identified for inclusion in the study. All selected districts (*n* = 8) had experienced A(H5N1) outbreaks. A total number of 24 villages were selected. The average number of persons per village was approximately 13.000 (range: 3.472–29.342); this corresponds with an average of 2.800 households per village. In each village the team would first meet with the village mayor (*el Omda*) or the head of the village council (*Sheikh el Balad*) to explain the aim of our study and to request for facilitation. During this informal meeting an assessment took place whether or not these individuals would be appropriate key informants. As the main purpose of the key informant interview was to inform a wealth ranking exercise the research team made sure that the selected key informant knew the population of the village. The key informant’s suitability and knowledge were carefully assessed by means of questions that related to the key informant’s familiarity with all social and wealth classes in the village, common indicators of wealth and poverty and issues affecting socio-economic status and well-being of community members. All key informants had public functions such as the village mayor or the head of the village council but key informants also included the village religious leader, senior teacher of the village primary school, director of the community development association or director of the agricultural cooperative. If the research team had any doubt about the suitability of the potential key informant alternative appropriate key informants would be selected based on referral by the village mayor.

A total of 24 key informant interviews were done, one in each study village. The interview took approximately 30 to 45 min on average. The main criteria used by key informants to differentiate between households were: ownership of land (and size of land), type of income source(s) such as seasonal or casual labour vs. fixed employment, marital status, ownership of large ruminants, education level, and receiving alms (*zakat*). A detailed list of common indicators was developed for each wealth group based on the 24 key informant interviews, see Additional file 2. Indicators mentioned unanimously by each key informant for a specific wealth group were identified. In this way, four standardized key characteristics/assets were identified across all villages; these included entitlement to alms, ownership of land, university education and fixed employment. Categorization by the key informant was verified for each household. Key characteristics of the household collected during the household interview were crosschecked against the list of common wealth and poverty indicators presented in Additional file 2, and compared to the categorization of the key informant. In no case did this yield reason to change the wealth group allocation of households by the key informant.

In addition, to establish credibility and reliability of the wealth indicators, the wealth classes were corroborated with different stakeholders within each village setting. The most common criteria used by key informants to establish household’s wealth status have also been identified in other studies as determinants of wealth [40, 41]. For example Croppenstedt [40] concluded that the poorest households in Egypt rely on casual employment and transfers (such as alms and social welfare pensions), while formal wage employment is associated with better-off households. He further stated that education is the key factor in determining wage employment and returns and these returns increase with more education. Household per capita income is positively correlated with landownership and landownership was associated with the two highest income quintiles. Lastly, female-headed households are particularly disadvantaged and can often be found among the lowest income quintiles.

The majority of key informants divided the population of their village into four different wealth categories: very poor, poor, medium and rich. It was decided to focus on the three lowest levels, excluding the rich. Key informants were then asked to identify households of each of these three different wealth groups. In this manner wealth ranking was used to stratify the sample at the household level. These households would then be visited by the team and asked to participate in an indepth household interview. Initially 144 households were approached of which two respondents indicated that they didn’t want to participate due to time constraints. Another two participants could not complete the entire interview and these two were omitted from the sample frame leaving a total of 140 respondents who completed the entire interview. These 140 households included 44 very poor households, 48 poor households and 48 medium-wealth households. Key informants also helped in allocating between five to seven participants for the group interviews, in which a mix representing different wealth levels participated. The duration of the household interviews as well as group discussions was usually between 60 to 90 min. Data collection took place during the months of September, October and November 2010.

During the analysis process the wealth groups were further stratified into subsets. Stratification was based on data collected during the household interview. Within the very poor, poor and medium wealth groups, the top level and bottom level of households were identified, thus creating two subsets within each wealth group. While the wealth ranking in the field divided the study group into three broad categories, the subsequent stratification during the analysis identified subsets of the very poor, poor and medium wealth groups based on a combination of specific indicators that were locally recognized as indicators of poverty or wealth. The initial phase of determining the subsets within the wealth groups yielded a wide variety of single and double item subsets. After initial testing and analysis, it was decided that the two-item subsets performed best in identifying those households at the top and bottom level of each wealth group. As we used two criteria, this meant that several households (*n* = 59) could not be included in the subsets. The subdivision within the very poor and poor wealth groups were based on education levels and marital status while subdivision among the medium wealth group was based on the size of land and the education level of the respondent. A total of 81 respondents matched the criteria described above and as mentioned in in Table 1.

**Table 1:** Description of subsets

Subset	Wealth group	Description of subset	Number of respondents
1	Very poor	Widowed or divorced & no education	13
2	Very poor	Married and some education	10
3	Poor	Widowed or divorced & no education	14
4	Poor	Married and some education	12
5	Medium	Up to primary education and ≤ 0.21 ha	22
6	Medium	Preparatory or higher education and > 0.21 ha	10
Total			81

The division into subsets enabled a more intricate understanding of how marriage and widowhood, education levels and access to resources influenced a wide range of issues such as vulnerability to A(H5N1), risk perceptions and preventive behaviours.

### The components of the risk index

Based on the existing literature, the CRI was composed of the following seven elements: poultry sales, previous flock exposure, human risk probability, sense of control over the disease, preventative actions taken, level of household food insecurity, community norms toward certain handling and disposal practices. These elements will be discussed in more detail in the subsequent sections.

#### Poultry sales

Live bird markets are well known A(H5N1) risk hotspots [2, 14, 19, 20, 42, 43]. Therefore, women or their household members either selling their birds directly at live bird markets or indirectly to traders have increased risk of both exposure to and subsequent transmission of A(H5N1).

#### Previous exposure to a(H5N1)

Previous outbreaks in household flocks may be indicative of A(H5N1) endemicity and less effective biosecurity measures being applied by the household. Furthermore A(H5N1) outbreaks in poultry increase the risk of human A(H5N1) infection [44]. Outbreaks were identified where the respondent reported experiencing high mortality rates in her poultry due to A(H5N1) infection during the years 2006 to 2010. Information thus depended on self-reporting and the perceptions of the respondents and it is acknowledged that this method can be flawed as respondents could have misinterpreted symptoms of other diseases (Newcastle disease in particular) as symptoms of A(H5N1). However information provided by the respondents, particularly on the symptoms observed during perceived outbreaks of A(H5N1), was crosschecked by the two female enumerators who were both well-experienced veterinarians.

#### Low perceived human risk

Risk perception levels were assessed by a three-point Likert-type scale using the following conditional statement: “*Imagine this village is infected with A(H5N1) in poultry*”. Respondents were then asked “*How likely would it be that you or your family members would be infected with A(H5N1)?”* Answer options were given as: “*not likely*”, “*somewhat likely*” and “*very likely*”. The ratings were used to assess the perceived A(H5N1) risk in terms of perceived likelihood of human infection (self or household members). To enhance understanding, questions regarding risk perception were contextualised as much as possible. For example, a time frame was included (i.e. respondents were asked to imagine an infection in poultry happening now), a setting (in case of an outbreak in this village) and risk object, i.e. the individual and her household members as opposed to the general population. Clearly respondents who perceived the likelihood of human infection to be low are likely not to engage in any self-protective measures thereby putting these respondents at a higher risk of infection.

#### Low sense of control over poultry infection

Qualitative content analysis was used to identify underlying rationales and beliefs pertaining to specific A(H5N1) risk levels. First the narrative information collected was categorized into broad themes identified by examining the transcribed interviews. As such, when respondents were asked to explain their perceived level of flock infection probability, four broad themes emerged: a) control practices, b) beliefs, c) exposure and, d) contact levels. Secondly, component topics underlying these themes were identified. Exposure, for example related to statements made by the respondents that included exposure or previous experience with A(H5N1) outbreaks. Topics included: “*previous outbreak in own flock*” and “*observed outbreak in neighbour flock*” The broad category of beliefs related to responses that implied certain beliefs, this included beliefs about the controllability of the disease, beliefs about susceptibility of poultry birds or the mode of transmission of the disease but also about the role of God in determining A(H5N1) infection. Specific topics identified under this theme were: “*bird flu is airborne”* or “*bird flu is spread by wild birds*”, “*God’s will*”, and “*bird flu can’t be controlled*”.

Any fatalistic notions as well as perceptions that indicated a sense of lack of control were noted. Perceptions of flock infection that indicated a locus of control outside of the respondent such as the particular belief that flock infection depends on God’s will are detrimental to A(H5N1) control and need to be targeted.

#### Lack of precautionary actions

Biosecurity is defined as measures taken to reduce the risk of introduction and spread of the A(H5N1) virus [17]. These biosecurity measures are based on the principles of bioexclusion and biocontainment [17]. With this in mind respondents were asked what kind of actions they had taken to protect themselves and their household from getting infected and the actions taken to prevent their flock from getting infected. For each of these two categories (human preventative behaviour and poultry preventative behaviour) the number of preventative actions weres counted. Next, the actions taken by each household were examined and only those mentioned in the biosecurity literature as being efficient were counted.

Those that engaged in two or less effective precautionary actions relevant for preventing poultry and/or human infection were deemed more vulnerable to A(H5N1) outbreaks while being more likely to spread the disease.

#### Food insecurity

At the household level food insecurity may lead to risky coping behaviour increasing the risk of A(H5N1) infection and spread such as consuming dead birds, sale of sick birds and not reporting sick birds or hiding birds in the house to avoid culling [8, 45]. A quantitative approach was used to construct the food insecurity score. The scale created for this study closely followed that of Blumberg et al. [46]. However, while Blumberg et al. [46] only addressed financial access to food, the present food insecurity component consisted of four elements prioritised in the literature: financial access, physical access, utilisation and socio-cultural dimensions. Responses were recorded by means of a closed-format binary scale (yes/no). Each food security component was then scored with zero or one: zero indicating that no problems and disruptions had been experienced by the household, and one indicating problems with the specific component of food insecurity in the past year. The sum of affirmative responses comprised a household’s raw score on the scale. The minimum score for each household would be zero (i.e. households had not experienced any disruptions across the four food security components in the past year) and a maximum score of four (indicating that the household had experienced disruptions across all four food security components).

The scoring helped to develop a scale from zero to four in which households on one end of the scale (four) could be considered more food insecure than those on the other end of the scale (zero).

#### Community norms detrimental to a(H5N1) control

Group norm has the power to influence individual decision-making [20]. Group or community norms regarding A(H5N1) prevention and control were assessed through focus group discussions. In each village one focus group discussion was held (*n* = 24). Participants were asked to rate five statements on a three-point Likert-type scale according to their notion of the acceptability of the action described. The five statements were as follows:

1. *“Is having your poultry scavenge freely in the street: acceptable, somewhat unacceptable, or unacceptable?”*
2. *“Is throwing dead birds and the remains of slaughtered birds in the street: acceptable, somewhat unacceptable, or unacceptable?”*
3. *“Is burying dead birds: acceptable, somewhat unacceptable, or unacceptable?”*
4. *“Is slaughtering sick birds to consume: acceptable, somewhat unacceptable, or unacceptable?”*
5. *“Is not reporting your birds infected with A(H5N1) to the local vet or village leader: acceptable, somewhat unacceptable, or unacceptable?”*

The group members rated the five statements collectively. Usually several group members would respond or engage in a discussion with their peers after the interviewer had read the question. As the group sizes were relatively small, the note taker of the group discussion was able to note the response of each group member in most cases. The interviewer would then solicit answers from those group members (if any) who had not spoken. The collective group rating was either based on consensus of the group or if no consensus was reached through taking the majority of ratings as the collective group answer.

Those communities where risky behaviours are generally accepted are least likely to have a positive effect on individual behaviours. Thus those communities where group norms indicated any of the following activities were acceptable were given a score of 1: scavenging of poultry in the village, throwing dead birds on the street, consuming sick birds and not reporting an outbreak or not burying dead birds.

### The composite risk index

The components of the Composite Risk Index are presented in Table 2.

**Table 2:** Composite risk index components

CRI loci	Factors	Score
Perception loci	Low human risk	1
Perception loci	Low sense of control	1
Perception loci	Community norm	1
Behaviour loci	Lack of precautionary action	1
Behaviour loci	Poultry sales	1
Predisposing loci	Previous exposure flock	1
Predisposing loci	Food insecure	1
Total		7

The maximum score for the CRI was seven for each respondent. The criteria for the index were as follows: those households who sold poultry birds received a score of one as those selling poultry were deemed more likely to spread the disease. Households having experienced one or more self-reported outbreaks received a score of one. Respondents who viewed the human risk probability to be low were deemed to be in need of subsequent awareness raising campaigns as were those respondents who engaged in only two or less correct preventative actions relevant for preventing human and/or poultry infection (also accorded a score of one). Perceptions of flock infection that indicated a locus of control outside of the respondent such as the particular belief that flock infection depends on God’s will were also given a score of one. The acceptability of particular strategies was also scored i.e. those villages where the collective norm noted any of the following activities was acceptable were given a score of one: scavenging of poultry in the village, throwing dead birds on the street, consuming sick birds and not reporting an outbreak or not burying dead birds. Finally, all households receiving a three or four as a Food Insecurity Score were deemed more vulnerable to the effects of A(H5N1) and therefore given a score of one.

Justification for the weighting of the index is as follows. Each of the factors in the attitudinal loci i.e. low human risk, low sense of control and community norm were each given a score of one. Core factors as part of the behaviour loci were also given a score of one i.e. lack of precautionary measures and poultry sales. Finally, factors in the ‘predisposing’ loci i.e. previous exposure to A(H5N1) and food insecurity were also given a score of one in the following equation:

$$ CRI= Perception\ Loci(3)+ Behaviour\ Loci(2)+ Predisposing\ Loci(2) $$

As described above, given that perceptions regarding animal disease drive behaviour this locus contained the largest number of factors and therefore a priori importance to the index. Consequently, the lack of precautionary or preventive behaviours toward the disease in addition to poultry sales were accorded a top score of two. The final portion of the index contained critical physical predisposing factors such as exposure to a prior outbreak and household-level food insecurity. By creating a linked scoring system, the CRI can be easily adapted to other geographies/nations.

### Statistical analysis

Continuous variables were compared using Independent-samples T-Test or One-way ANOVA. Categorical variables were analysed using Chi-square tests and Fisher’s exact tests. For all analyses, significance levels were two-tailed, and a *P* value of 0.05 was considered significant. All analyses were performed using SPSS Version 22.0 (IBM Corp, USA).

## Results

Eighty-one respondents (61.3%) reported having experienced one or more A(H5N1) outbreaks. Over one-third of the very poor households (35.7%) experienced multiple A(H5N1) outbreaks. An One-way ANOVA test indicated that the very poor experienced a higher number of self-reported outbreaks on average during the study period (m = 1.24, ±1.14) compared to the poor (m = 0.91, ±1.05) and medium (m = 1.02, ±1.19) households, however this difference was not significant, *p* = 0.395. Across the subsets, the difference in the number of self-reported outbreaks was more striking, see Table 3.

**Table 3:** Self-reported A(H5N1) outbreaks in household flocks by subsets

Subset	No outbreaks (%)	One outbreak (%)	Two or more outbreaks (%)	Number of self-reported A(H5N1) outbreaks (average)
1	15.4	38.5	46.2	1.6
2	50.0	30.0	20.0	0.8
3	35.7	35.7	28.6	1.1
4	58.7	33.3	8.3	0.5
5	45.0	20.0	35.0	1.2
6	37.5	62.5	0.0	0.6

Households in Subsets one, three and five had experienced approximately two times more A(H5N1) outbreaks in their flock compared to their better-off counterparts (subsets two, four and six) in the same wealth group. Although an One-way ANOVA test indicated that these differences were not statistically significant, *p* = 0.155, the findings do show a trend of higher vulnerability to A(H5N1) flock infection among widowed or divorced women with little or no education or agricultural land.

The results from the CRI scores across governorates and study villages are detailed in Additional file 3.

The most southern located governorate of Souhag had the highest CRI score (4.12). Food insecurity is particularly prevalent among households in this governorate, contributing in part to the high CRI score. Overall, nine villages had CRI scores of 4.0 or greater indicating their risk to A(H5N1) infection.

Across the study set five villages had an awareness raising campaign. An Independent samples T-test was run to determine if the average CRI score of respondents was different between villages where awareness campaigns had taken place and where no awareness campaigns had taken place. Mean CRI score for villages where awareness raising had taken place (3.75) and villages where no awareness raising had taken place (3.67) was not statistically significant, *p* = 0.844. Having had an awareness raising campaign was thus not associated with significantly reduced risk of flock infection.

With regards to social norms, overall scores indicate that slaughtering sick and dead birds and consuming them is considered the least acceptable, followed by throwing dead birds and slaughter remains on the street, not reporting if birds are infected, having poultry scavenge freely in the street and lastly burying dead birds. No differences were found with regards to community norms in villages where awareness campaigns had taken place vs. those where no awareness campaigns had taken place. The most interesting results relate to high percentage of respondents who offered that such behaviours around sick or dead birds were acceptable. For example, over one-third of study participants ranked poultry scavenging as acceptable while 20% noted that not reporting an A(H5N1) outbreak was equally OK. Despite 75% of the study group noting that burying dead birds was ‘acceptable’, the most common disposal method for dead birds was throwing them in or near canals or on garbage heaps.

Respondents in three of the CRI high score villages (Nagu Aba Awad, Nazlat Fareg Mahmoud and El Robeiat) had been exposed to an awareness campaign. Interestingly, Nazlat Fareg Mahmoud had collectively among the highest score for lack of preventive actions and also scored highest on low sense of control i.e. the majority of respondents expressed flock infection was beyond their control. Thereby, the composite index can reveal particular areas for improvement in subsequent awareness campaigns.

**Fig. 1:**
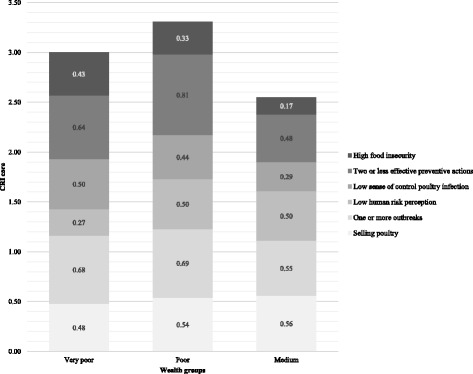
CRI scores among wealth groups

The CRI scores for the wealth groups are presented in Fig. 1.

As Fig. 1 illustrates, medium wealth level households had the lowest CRI score (2.55). Interestingly, the poor as opposed to the very poor had the highest CRI score, 3.31 and 3.00 respectively. This high score can mainly be attributed to the high score (3.64) of the uneducated widowed or divorced subset within this wealth group.

**Fig. 2:**
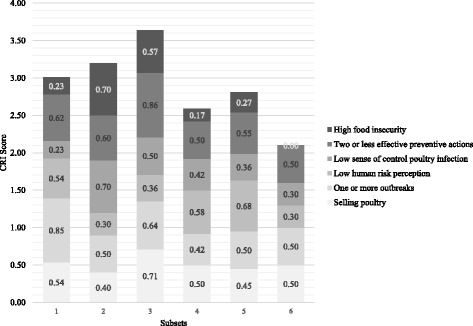
CRI scores among subsets

The CRI scores for the subsets are presented in Fig. 2.

Subset three and five scored persistently higher compared to their better-endowed counterparts within the same wealth group (i.e. subset four and six). The difference between subsets three and four was striking. Subset three had the highest component score for selling of poultry and lack of precautionary behaviours.

A Q-square test indicated that higher education levels (i.e. secondary and higher) in particular were found to be associated with a range of effective biosecurity measures, see Table 4.

**Table 4:** Associations between higher education and effective preventive biosecurity measures

Biosecurity measures	*P* value	Strength of association (Phi or Cramer’s V)
Hand washing after handling poultry	0.027	0.200
Changing shoes and clothes before attending to poultry	0.021	0.208
Vaccinating poultry	0.000	0.390
Keeping wild birds away from flock	0.004	0.256

Lack of education was particularly associated with lack of precautionary actions, *p* = 0.009 (Phi = 0.236, *p* = 0.009). Subset six had the lowest CRI score, thus indicating lower risk of A(H5N1) infection compared to the other groups. This can be attributed to relatively low scores on each of the individual components but in particular food insecurity and risk perceptions (Fig. 2). None of the respondents were food insecure (score 0.0) and as the analysis pointed out, food security is particularly associated with wealth and higher education levels. Only a minority expressed that A(H5N1) infection of their flock was outside of their control (0.30) and few believed the likelihood of human infection to be low (0.30).

Overall the very poor and poor as well as subsets where women were widowed or divorced with low education levels were likely more vulnerable to food insecurity and A(H5N1) outbreaks in their flocks. These groups tended not to take effective precautionary actions and also had a tendency to express fatalistic beliefs. For example the belief that human infection depends on God’s will is significantly associated with the poor and very poor wealth level, *p* = 0.039 (Cramer’s V = 0.245, *p* = 0.039). The belief that A(H5N1) virus could be transported through air for large distances, i.e. from one governorate to the other, and therefore nothing could be done about it was significantly associated with very poor households, *p* = 0.004 (Phi = 0.274, *p* = 0.004).

## Discussion

Controlling A(H5N1) in Egypt is an urgent priority of the Government. However, the results of the study illustrate that ultimately stamping out the disease may rely on more targeted interventions and approaches that account for a range of abiotic drivers of disease from socio-economic status to perceptions and beliefs regarding causality and sense of control over the disease itself. This is in line with Paul et al. [20] who concluded that a multidisciplinary approach is needed to study common practices and underlying believes and rationales to be able to develop policies that take more account of the realities on the ground. The implementation of effective biosecurity measures within resource poor contexts depends upon careful consideration of the interests of poultry farmers [5, 17, 19, 21, 45].

Application of the Composite Risk Index points to communities where vulnerability to A(H5N1) infection is likely to be high. Vulnerability to A(H5N1) infection at the community level is multifaceted and complex and as the study illustrated, based on a variety of factors. Tools which enhance targeting and identifying areas where values, norms and attitudes may inhibit control efforts at the outset are likely to improve disease outcomes at the community level.

Many authors have commented on the fact that, although national A(H5N1) awareness raising efforts may have been successful in increasing awareness and knowledge of A(H5N1), the gap between knowing what to do and applying this knowledge remains large [20, 47]. Equally this study showed that prior awareness raising campaigns had not resulted in a significant increase in effective precautionary behaviours. This is likely explained by a discrepancy in focus and priorities between government officials and poultry producers. This study pointed to the fact that concerns of respondents related more to loss of food security and income as a result of A(H5N1) outbreaks and associated control measures and less to human infection, while the latter had been the main concern of the Egyptian government. Such discrepancies with regard to priorities of national governments and backyard poultry producers have also been described by Velasco et al. [18] and Paul et al. [20], and have been commented on as a serious barrier for effective A(H5N1) prevention and control.

In Laos, training on A(H5N1) prevention is usually given to men in urban locations while the target group is mainly female, live in remote areas and are often of ethnic minority who do not speak Lao [18]. Moreover such training commonly focuses on technical aspects while lacking consideration of underlying beliefs and rationales that influence risk perception and protective behaviours or lack thereof.

Therefore, future awareness raising efforts need to account for these concerns with special attention to the poor and widowed/divorced subsets of household poultry keepers who proved to be particularly vulnerable to A(H5N1) outbreaks. Velasco et al. [18] also described how the poor and women in particular face difficulties in accessing information and how lack of education increased their risk of infection.

Widowhood was unanimously regarded as an indicator of poverty among the study participants. While women living in households with access to land and with higher education levels were recognized as part of the ‘better-off’ at the community level. Therefore uneducated, widowed women had a very different experience than married educated women. To accommodate these differences, a range of subsets were created that combined a range of recognized demographic, social and wellbeing indicators related to poverty and these were used along with the broader wealth groups to look at how these elements may inform risk behaviour and perceptions, livelihood activities and food security. The subset approach described in this article enabled a more intricate understanding of how marriage/widowhood, education levels and access to resources influenced a wide range of issues such as vulnerability to A(H5N1), risk perceptions and preventive behaviours.

We found that education was related to the application of effective biosecurity measures. Similarly several authors have described how education had a positive effect on avian influenza related knowledge as well as preventive measures in Thailand [18, 20], Taiwan [48], Vietnam [49], Nigeria [50] and China [51]. Among the study set poverty was linked to lack of education and a lack of effective precautionary behaviours, thus making the poor and their poultry even more vulnerable to A(H5N1) infection.

Among the cockfighting community in Thailand “*strategies to avoid HPAI surveillance and control measures were actually collectively organized, [and] relied on strong social bonds.”* ([20]: page 113). In our study we found that even if individuals perceive certain behaviours such as wearing protective clothes or burying dead birds as reducing the risk of infection, group norm (through mockery among others) may impede individual beneficiary precautionary action. Such cultural related group norms can thus prevent implementation due to feelings of shame and embarrassment even among those that do understand and regard the practice as beneficial. This is particularly important in settings where social standing and acceptance are highly valued and important.

## Conclusions

The CRI points to high-risk communities but also helps in identifying key areas within the community that need strengthening. These could relate to community norms impeding implementation of biosecurity measures or prevalence of outbreaks.

The CRI has been developed keeping in mind that its application in the field should be practical and simple. Thereby keeping time and data requirements to a minimum while still being comprehensive enough to account for relevant social, economic, epidemiological and psychological factors both at the individual and community level contributing to vulnerability to A(H5N1) infection in both poultry and humans.

However, overall the tool was created with the budgetary and time constraints of field staff on the ground by focusing on seven indicators for which a short questionnaire addressed at the household level will suffice. In Egypt, some of the data requirements for the CRI might be readily available within the Central Agency for Public Mobilization and statistics (CAPMAS) or the village information units. In some instances, reliable information on A(H5N1) outbreaks at the community level might be available and this information could replace the household level data on self-reported A(H5N1) outbreaks, thereby reducing data requirements.

The core themes of risk perception, epidemiology and food security are relevant for a wide variety of other infectious diseases and the indicators used within these core themes can be easily adapted to suit local circumstances across a range of geographies.

## Additional files

Additional file 1: Questionnaires used for the present study. This file shows the questionnaires and interview guides used for the key informant interview, the household interview and the focus group discussions. The original questionnaires were used as part of the PhD thesis of the first author. The questionnaires presented here show only the parts that are pertinent to the presented data in this manuscript. (DOCX 28 kb)

Additional file 2: Classification of wealth groups. This file shows the results of a wealth ranking exercise. It summarises the criteria used by 24 key informants to distinguish between different wealth groups. (DOCX 18 kb)

Additional file 3: Composite Risk Index score across governorates and study villages. This file shows the individual component scores as well as the overall composite risk scores for each study village and governorate. (XLSX 10 kb)

## Abbreviations

A(H5N1): Highly Pathogenic Avian Influenza (HPAI) subtype H5N1
CAPMAS: Central Agency for Public Mobilization and statistics
CRI: Composite Risk Index
FAO: Food and Agriculture Organization
VAM: Vulnerability Analysis and Mapping
WFP: World Food Programme
