# Supplementary material for: Predicting risk of avian influenza a(H5N1) in Egypt: the creation of a community level metric
Source: BMC Public Health. 2018 Mar 21;18:388. doi: 10.1186/s12889-018-5288-x (PMC5863456; doi:10.1186/s12889-018-5288-x)
Supplement: Supplementary file 1 — Questionnaires used for the present study. This file shows the questionnaires and interview guides used for the key informant interview, the household interview and the focus group discussions. The original questionnaires were used as part of the PhD thesis of the first author. The questionnaires presented here show only the parts that are pertinent to the presented data in this manuscript. (DOCX 28 kb) [file 12889_2018_5288_MOESM1_ESM.docx]

**Guidelines for interviewers and note takers**

Before starting the key informant interview, household interview and group interview please adhere to the research procedures agreed upon during the last day of the training course.

These procedures are summarized below:

1. Provide an explanation to people participating in the interviews of who we are and why we are doing this research.
2. Emphasize that we are neutral and not representing the government or local authorities. As researchers we should be non judgemental and non-directive.
3. Please explain that participation is voluntarily and does not involve immediate benefits. Therefore, respondents should be asked if they have time to participate and if they agree to participate. Formal oral consent should be obtained after introduction of the research team to the potential respondent and after the purpose of the research has been explained.
4. Please explain that information will be treated with confidentiality and names will not be reproduced in any work or publication resulting from this study.
5. Remain calm and friendly during the interview.
6. At the end of the interview give people some time to ask questions or add things they haven’t been able to mention during the interview.
7. Thank people for their time and participation before leaving.

**Key informant interview**

Date:

Interviewer:

Note taker:

Governorate:

District:

Village:

Name of respondent:

Function in the village:

Male/Female

**A) Guidelines for wealth ranking exercise**

1) Can you please describe the characteristics that are generally considered to be representing wealth, resilience and well-being in this village?

2) Please also describe those characteristics, which are associated with poor and vulnerable households in the village.

3) What are the key characteristics of a typical **very** **poor** household in the village? You might want to consider the following issues (*use the following prompts*):

- What they own (assets)
  - Land
  - Livestock
  - Other assets
- What they do in order to generate income (main source of income). If possible an estimate of their average monthly income
- How dependent they are on poultry keeping for food and income,
- Their level of education
- Their levels of savings and their sources of credit
- Marital status

4) What are the key characteristics of a typical **well-off** household in the village? You might want to consider the following issues:

- What they own (assets)
  - Land
  - Livestock
  - Other assets
- What they do in order to generate income (main source of income). If possible an estimate of their average monthly income
- How dependent they are on poultry keeping for food and income,
- Marital status

5) Which different group(s) fall between these two extremes (very poor households and well-off households) and what are their characteristics, i.e. what criteria are used to differentiate between households of different wealth?

Group 1:

Characteristics

Group 2 (optional):

Characteristics

**B) Avian Influenza**

6) What did people in this village do with sick or dead birds during the HPAI outbreaks? (*Possible probes: throw birds in river or on street, slaughtered them and store them in a freezer for consumption, sold them at the market, buried them*?)

7) What official laws and rules exist to govern the keeping, trade and slaughter of live birds in this village, are they enforced and if so how?

8) What unofficial/social rules exist in the village regarding the keeping, trade and slaughter of live birds in this village?

9) Have any avian influenza education/awareness raising campaigns taken place in this village?

**Household interview**

Date:

Interviewer:

Note taker:

Governorate:

District:

Village:

Name of respondent:

Age:

Marital status:

Education level:

Household size: Male adults (18 and above): Female adults:

Children: Girls: Boys:

**A) Income sources and assets**

1) What are the main income sources of your household?

2) Do you receive any outside support in the form of zakat, government social pensions or support from well-off villagers?

3) In addition to your current income generating activities what would be other options for you to gain income and/or payment in kind (of food items or livestock for example) in case the need arises?

4) Can you indicate the most important issues that have been affecting you or your family’s economic situation, diets, well-being? Please rank them from most bad to least bad.

5) Please indicate the type and number of livestock you currently own?

(*Use following prompts*)

Buffalo:

Camel:

Cow:

Sheep:

Goat:

Poultry birds:

Other (please specify):

6) Do you sell live birds? If so, can you provide an estimate for the contribution of poultry income to total household income?

7) Do you own or rent agricultural land, if so please indicate the size in kirat.

Owned: kirat

Rented: kirat

**B) Avian Influenza**

8) How many times has your flock been infected with avian influenza since the first outbreaks appeared in Egypt in 2016?

(*Note to interviewer: please ask for detailed symptoms and crosscheck with your own professional knowledge, when in doubt please make notes*)

9) What did you do with your sick or dead birds when the last outbreak occurred?

**C) Attitude & risk perception towards HPAI**

10) Imagine this village is infected with avian influenza in poultry, how **likely** would it be that you or your **family members** would be infected with avian influenza? Please use the following ratings: 1= not likely; 2= somewhat likely 3=very likely

11) Please explain to us why you indicated 1, 2 or 3?

12) Imagine this village is infected with avian influenza in poultry (*other than own flock*), how **likely** would it be that your **poultry flock** would be infected with HPAI? Please use the following ratings: 1= not likely; 2= somewhat likely 3=very likely

13) Please explain to us why you indicated 1, 2 or 3?

14) Please indicate the actions that **you and the members of your household** have taken in order to prevent infection with avian influenza.

15) Please indicate the actions taken to prevent infection of your **birds** with avian influenza.

**D) Food security**

16) Do you have any problems assessing any food items or other necessities to prepare food (e.g. are there enough markets or food stores within walking distance)? Yes/no

- If yes, indicate what the problem was.

17) Have you experienced a situation in the past year where you were not able to obtain necessary food items for normal everyday meals due to lack of money? Yes/no

- If yes, indicate what the problem was.

18) Have you experienced a situation in the past year where you were not able to obtain necessary food items to offer to guests or to provide during socio-cultural/socio-religious functions? Yes/no

- If yes, indicate what the problem was.

19) Have you changed your and your household’s diet in recent years? Yes/no

- If yes, what was the reason and how exactly have you changed the diet?

**Focus group discussion**

Date:

Interviewer:

Note taker:

Governorate:

District:

Village:

Number of respondents:

**A) Attitude and social norm**

1) What do people in this village normally do with sick or dead birds?

2) Can you indicate if the following practices are: acceptable=1, somewhat acceptable=2, or unacceptable=3

- Having your poultry scavenge freely in the street.
- Throwing dead birds and slaughter remains on the street in the village.
- Burying dead birds.
- Slaughtering sick birds and consuming them.
- Not reporting (to the village vet or village leader) if your birds are infected with avian influenza.
