# Supplementary material for: Predicting risk of avian influenza a(H5N1) in Egypt: the creation of a community level metric
Source: BMC Public Health. 2018 Mar 21;18:388. doi: 10.1186/s12889-018-5288-x (PMC5863456; doi:10.1186/s12889-018-5288-x)
Supplement: Supplementary file 2 — Classification of wealth groups. This file shows the results of a wealth ranking exercise. It summarises the criteria used by 24 key informants to distinguish between different wealth groups. (DOCX 18 kb) [file 12889_2018_5288_MOESM2_ESM.docx]

| **VERY POOR**  **Income sources:**  * Men in the household have irregular (seasonal) income from working as a labourer in construction work or on agricultural land.  * Women may earn income from selling poultry and eggs or vegetables in the market.  * Children may contribute to the household income by working as petty traders (selling household items) or helping their parents working on the land of the richer households, or as some mentioned, by winning cash prizes for memorizing the Holy Koran.  * The very poor are entitled to receive Zakat.  * The poor and needy are entitled to a government “Sadat” pension which amounts to 50LE per month. This pension is for widowed, divorced or handicapped persons and people without formal employment. However persons within the target group are not always capable of applying for the pension, as it requires certain documents.  **Role of Poultry:**  * Poultry often forms the most important source of income for these households and the main source of animal protein  **Land ownership:**  * The very poor do not own agricultural land but may work on the agricultural lands of richer households.  **Assets and house:**  * The house is simple and built from brick stone, often the house is not owned but rented  * They do not own electrical appliances such as washing machines or refrigerators  * Some may have a small stove and a second hand black and white TV. paid on instalments  **Education:**  * Children attend primary school and sometimes secondary school but cannot afford the cost to send their children to College and University.  **Additional comments:**  This group often includes widowed, divorced and old people. | **POOR**  **Income sources:**  * Men in the household may have irregular seasonal work or work as governorate employee owning low but regular salary as office boy, soldier, policemen or railway worker, or they work in the municipality as guards in the health unit or watchman in a farm or mosque.  * Some men in the household earn income from selling vegetables, fruit and bread or earning income from irregular work.  * Women earn income by selling poultry and eggs.  * Men and women in the household may earn income from taking care of cattle for other people or by share owning a cow or buffalo.  * Children in these households may contribute to the household income by working as petty traders  * The poor and needy are entitled to a government “Sadat” pension which amounts to 50LE per month.  **Role of poultry:**  * Poultry often forms a very important source of income for these households and the main source of animal protein  **Land ownership:**  * Majority don’t own agricultural land but a minority could own some karats in agricultural lands from which they earn a nominal income  **Assets and house:**  * The house is simple  * They may have a TV and an old stove and refrigerator, some have an old washing machine  **Education:**  * Children attend primary and secondary  **Additional comments:**  This group may include widowed, divorced and old people. | **MEDIUM**  **Income sources:**  * Men in the household have regular work as Government employee earning a moderate income (at least LE1000/month).  * Some households own a cow or buffalo and they earn income by selling its milk  * Some households have male family members working abroad from which they receive financial support every month.  * Women may earn income by selling poultry and eggs or working as teachers, nurses or development workers in the Governorate or Municipality  **Role of poultry:**  * Poultry may provide a supplement to the total household income and forms an important source of protein but red meat and fish are also consumed regularly  **Land ownership:**  * Some own agricultural land from which they earn revenues from selling crops  **Assets and house:**  * The house is moderate and often owned by the household  * They own modern machines and electrical appliances like stoves, TV’s, refrigerators and washing machines  **Education:**  * The majority of households is able to send their children to College and University  **Additional comments:**  This group may receive remittances from family members working abroad |
| --- | --- | --- |
